# Prevalence and risk factors associated with malaria infection in children under two years of age in southern Togo prior to perennial malaria chemoprevention implementation

**DOI:** 10.1186/s12936-023-04793-y

**Published:** 2023-11-21

**Authors:** Shino Arikawa, Martin Kouame Tchankoni, Fifonsi A. Gbeasor-Komlanvi, Somiabalo P. Atekpe, Tinah Atcha-Oubou, Antía Figueroa-Romero, Augustin E. Fombah, Francisco Saute, Mohamed Samai, Clara Menendez, Raquel Gonzalez, Valérie Briand, Didier K. Ekouevi, the MULTIPLY consortium

**Affiliations:** 1 University of Bordeaux, National Institute for Health and Medical Research (INSERM) UMR 1219, Research Institute for Sustainable Development (IRD) EMR 271, Bordeaux Population Health Research Centre, Bordeaux, France; 2 Département de Santé Publique, Université de Lomé, Lomé, Togo; 3 Centre Africain de Recherche en Epidémiologie et en Santé Publique (CARESP), Lomé, Togo; 4 Ministère de la Santé, de l’Hygiène Publique et de l’Accès Universel Aux Soins (MSHPAUS), District Sanitaire du Haho, Notsé, Togo; 5 Ministère de la Santé, de l’Hygiène Publique et de l’Accès Universel aux Soins (MSHPAUS) Programme National de Lutte contre le Paludisme (PNLP), Lomé, Togo; 6 Barcelona Institute for Global Health, Hospital Clinic‑University of Barcelona, Barcelona, Spain; 7 College of Medicine and Allied Health Sciences, University of Sierra Leone, Freetown, Sierra Leone; 8 Directorate of Research and Training, Ministry of Health, Freetown, Sierra Leone; 9 Manhiça Health Research Center, Manhiça, Mozambique; 10 Epicentre, Paris, France

**Keywords:** Malaria infection prevalence, Prevention, IPTi, PMC, Children, Sub-Saharan Africa, Togo

## Abstract

**Background:**

Malaria remains the leading cause of mortality and morbidity in young children in sub-Saharan Africa. To prevent malaria in children living in moderate-to-high malaria transmission areas, the World Health Organization has recommended perennial malaria chemoprevention (PMC). Prior to piloting PMC implementation in southern Togo, a household survey was conducted to estimate malaria infection prevalence in children under 2 years of age (U2).

**Methods:**

A cross-sectional community-based household survey was conducted in the Haho district in the Togo Plateaux region. A three-stage random sampling method was used to select study participants aged 10–23 months whose caretakers gave informed consent. The prevalence of *Plasmodium* infection, defined as a positive rapid diagnostic test (RDT), was estimated with 95% confidence interval (CI). Clinical malaria was defined as having a positive RDT plus fever (≥ 37.5 °C) or history of fever in the last 24 h. Mixed-effects logistic regression models were used to assess the child’s, caretaker’s, and household’s factors associated with malaria infection.

**Results:**

A total of 685 children were included in the survey conducted January–February in 2022 (dry season). Median age was 17 months (interquartile range: 13–21). About 80% of the children slept under a bed net the night before the interview. Malaria infection prevalence was 32.1% (95% CI 27.7–37.0) with significant area variation (cluster range: 0.0–73.3). Prevalence of clinical malaria was 15.4% (95% CI 12.2–19.2). Children whose caretakers were animist (aOR: 1.71, 95% CI 1.19–2.46) and those living in mother-headed households (aOR: 2.39, 95% CI 1.43–3.99) were more likely to have a positive RDT. Living more than 5 km away from the nearest health facility (aOR: 1.60, 95% CI 1.04–2.44) and presence of two or more under-5-years children in the household (aOR: 1.44, 95% CI 1.01–2.07) were also associated with increased risk of infection.

**Conclusion:**

One-third of the children U2 who participated in this survey had malaria infection, thus PMC could be a promising strategy to reduce malaria burden in young children in Plateaux region. Reinforcement of outreach services and targeting the poorest households should be prioritized to reduce the inequity in malaria prevention in children exposed to the infection.

## Background

In recent decades, progress has been made to reduce malaria burden in high endemic areas. However, malaria remains a serious public health problem in Togo affecting a large number of people [1]. In 2019, 2.4 million malaria cases were reported in Togo, accounting for the country’s most endemic parasitic disease both in terms of morbidity and mortality. Children under 5 years of age (U5) take the highest burden, accounting for 36% of uncomplicated malaria cases, 58% of hospitalized cases, and 73% of malaria deaths [2, 3]. The National Malaria Control Programme (NMCP) has made tremendous efforts in scaling up anti-malarial interventions through its National Strategic Plans. To prevent malaria in children, the NMCP built its strategy around two pillars: (i) distribution of long-lasting insecticidal nets (LLINs) through routine medical visits to children under 1 year of age and (ii) seasonal malaria chemoprevention for children age 3–59 months in the northern part of the country where malaria transmission has a seasonal pattern [2].

In 2010, in the areas where malaria transmission is perennial, the World Health Organization (WHO) recommended intermittent preventive treatment of malaria in infants (IPTi) to those living in moderate-to-high malaria transmission areas where parasitic resistance to sulfadoxine-pyrimethamine (SP) is low [4]. IPTi involves administration of a full therapeutic course of SP, regardless of infection, delivered to infants through the Expanded Program on Immunization (EPI) at predetermined intervals corresponding to routine vaccination schedules. IPTi aims to clear current infection while preventing new ones for around 28 days during which time the drug concentration in the bloodstream slowly wanes [5]. Despite the body of evidence demonstrating its efficacy, safety, and cost-effectiveness, IPTi was only adopted as a national policy in Sierra Leone [5–11]. In mid-2022, the WHO renamed IPTi as perennial malaria chemoprevention (PMC) and recommended that PMC be provided to children beyond 12 months of age and that the strategy be adapted to the local epidemiological context [12]. To facilitate the introduction of this strategy and to inform subnational tailoring, a pilot implementation of PMC has been planned in southern Togo through the MULTIPLY project [13]. Prior to its implementation, a household survey was conducted to assess the prevalence of *Plasmodium* infection and associated risk factors in children under 2 years of age (U2), who are the target population of PMC.

## Methods

### Study design

A cross-sectional community-based household survey was conducted in the Haho district from January to February 2022. The Haho district is located in the Plateaux region approximately 100 km from the capital city Lomé. Malaria transmission in the area occurs along the year with peaks in two rainy seasons (March–July and September–October) [14]. Therefore, the NMCP does not consider the areas eligible for seasonal malaria chemoprevention implementation [2].

### Study population and sampling methods

The survey included children U2 who live in the district and meet the following criteria: (i) age 10–23 months (inclusive), and (ii) the child’s caretaker’s agreement to participate in the research by signing the informed consent form. To calculate the sample size, a design effect of 2, 5% precision and 10% non-response as well as the malaria prevalence estimate in Togo in 2017 (28%) were assumed [15]. Based on these assumptions, minimal sample size necessary for the survey was estimated as 682 children. To select the children for the survey, a three-stage cluster sampling method, adapted from the Malaria Indicator Survey and the EPI sampling method, was used [16, 17]. In the first stage, clusters were randomly chosen from a comprehensive list of villages within the district using probability proportional to size. In the second stage, households were randomly selected from a full list of households based on the household counting exercise undertaken by the enumerators. Finally, in the third stage, a random selection of eligible children was done in selected households. It was assumed that fieldworkers could interview the caretakers of 12 children in one cluster. Thus, the number of clusters to be surveyed was determined as 57 (682/12).

### Data collection

A standardized questionnaire was administered through the REDCap mobile application to collect socio-demographic and behavioural data on the children (age, sex, vaccination/vitamin A uptake, bed net use), caretakers and household heads (age, relationship with the child, educational status, literacy, income, religion, ethnic group) as well as caretakers’ knowledge and perceptions about malaria [18]. Information on the household characteristics (the number of children U5 living in the household, distance to the nearest health facility, geolocation) was also obtained. Eligible children underwent a malaria rapid diagnostic test (RDT) regardless of symptoms. Those who were tested positive were treated free of charge according to the national guidelines and referred to a health facility in case of severe malaria.

Data collection was carried out by 25 teams of two health professionals who received a 4 day training session that covered research protocol, informed consent procedures, questionnaire administration using tablets, household and child selection, and the use of RDTs. Each team was assisted by a community health worker (CHW) who facilitated access to the households’ members.

### Statistical analyses

To summarize quantitative and qualitative data, the median with interquartile range (IQR) and proportion with a 95% confidence interval (CI) were used respectively. Data were weighted to reflect the multi-stage cluster sampling design. Weights were calculated for each sampling stage while those in the third stage, namely the random selection of a child, were applied only to the data from the households having multiple eligible children. The prevalence of malaria infection was defined as a weighted proportion of children who was tested positive for malaria RDT among all children. Similarly, the prevalence of clinical malaria among all participating children was estimated as a weighted proportion of children with a positive RDT and history of fever (≥ 37.5 °C) within the past 24 h or on the day of the survey.

To assess factors associated with children’s malaria infection while accounting for correlation between clusters, mixed-effect logistic regression models were built with clusters included as a random effect. Factors tested in the models were selected a priori based on previous knowledge about predictors of malaria in children and adults [19–26]. Children’s age, divided into two categories at the median age (10–16 vs. 17–23 months), was included in all models irrespective of statistical significance.

First, the association between malaria infection and children’s characteristics including age, use of a mosquito net the night before the interview, immunization status, and vitamin A uptake was tested. Children’s immunization status was classified into three categories (fully immunized, partially immunized, or never immunized) according to the information available in the child’s EPI card or on the basis of the caretaker’s self-declaration if the card could not be located at the time of the interview. Fully-immunized children were those who have received all the routine scheduled vaccines by 9 months of age according to Togo’s national EPI schedule. If any of the vaccines were missed, children were considered as partially immunized. Vitamin A was recommended at the age of 9 months in Togo, thus vitamin A uptake was defined as binary (yes, no).

Second, the association between the child’s malaria infection and the characteristics of caretakers or household heads was investigated. In this model, the factors significantly associated in the first model (P < 0.05) as well as caretakers’ characteristics including age, marital status, educational level (no formal education, primary, secondary, or above), literacy (fully literate, partially literate, illiterate), ethnic group, religion (Christian, animist, other), income source (formal employment, self-employment, no income), and possession of child’s EPI card (yes, no) were included. The literacy level was defined by reading and writing exercises included in the questionnaire. Caretakers who were able to read and write the requested sentences without any difficulty were classified as “fully literate.” Those with difficulties in at least one of those exercises were defined as “partially literate,” and anyone incapable of reading or writing was considered as “illiterate.” In this model, the variables related to the characteristics of household heads such as his/her relationship with the child (mother, father, another family member), education level, and income source, were also included.

Finally, a model was built with all the variables significantly associated with the outcome in the previous model (P < 0.05) and the households’ characteristics including the number of children U5 (one vs. two or more) and the village’s location in terms of the distance from the nearest health facility (< 5 vs. ≥ 5 km).

P-value < 0.05 was considered as statistically significant in the final model. Statistical analyses were performed using the lme4 package in R version 3.6.2 (R Development Core Team, 2004) [27].

## Results

**Fig. 1:**
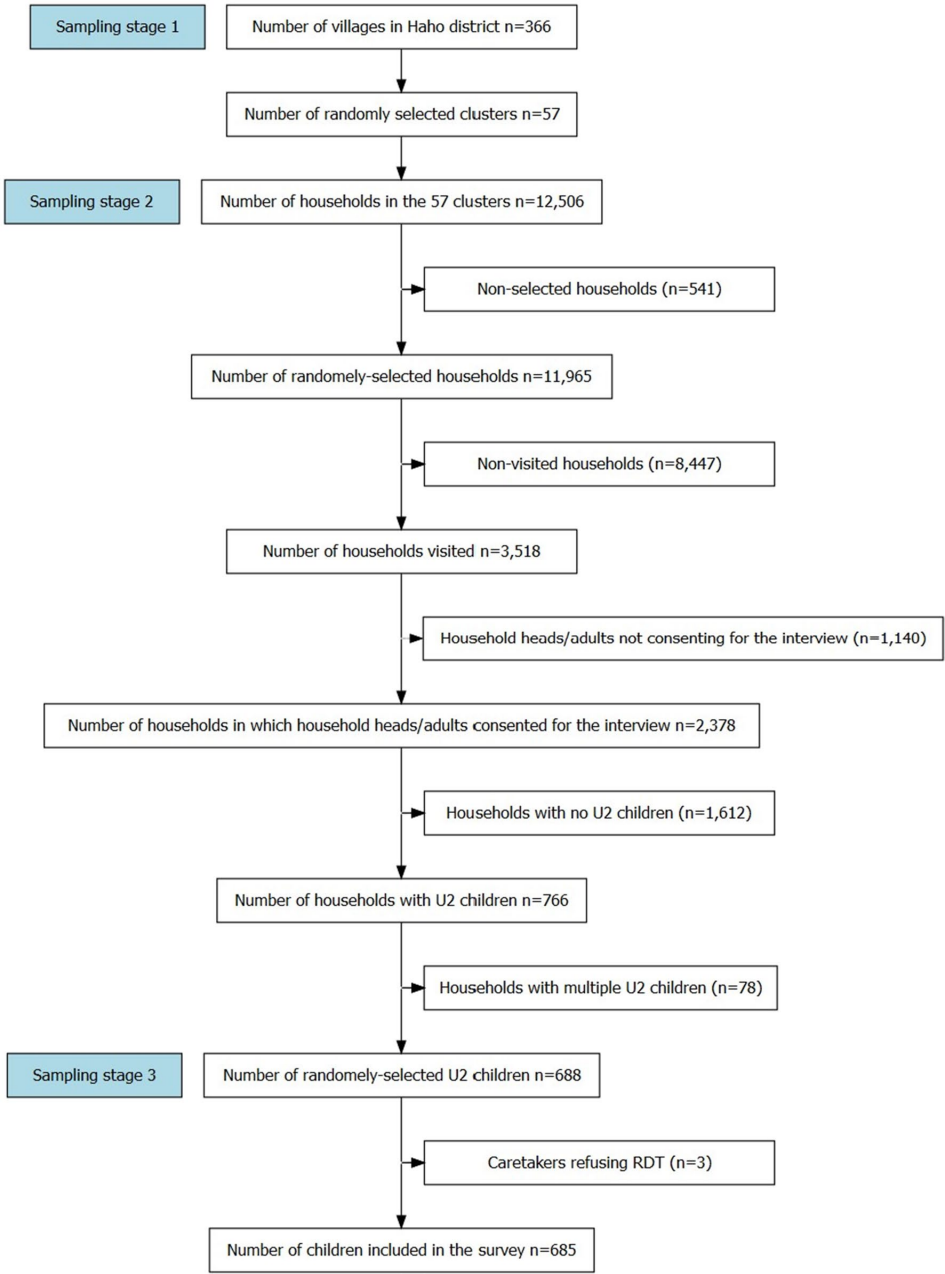
Flow chart of the survey population using 3-stage random sampling method

Based on the enumeration exercise, 12,506 households were identified in the 57 clusters. A total of 3518 households were visited and of 685 children were included in the survey (Fig. 1).

### Socio-demographic characteristics of the children, caretakers, and household heads

The children (48.1% girls) had a median age of 17 months (IQR: 13–21) (Table 1). The majority (79.1%) were declared to have slept under a bed net the night before the interview. Around 60% of the participants (58.7%) had received all the scheduled vaccines by 9 months of age and thus were considered to be fully immunized. Almost 14% have never been vaccinated. Vitamin A uptake was 61.3%.

**Table 1:** Description of the study population (N = 685)

Children	
Age (months), median (IQR)^1^	17.0 (13.0–21.0)
Age groups, n (%)	
10–16 months	302 (44.1)
17–24 months	383 (55.9)
Slept under a bed net the night before (Yes), n (%)	541(79.1)
Immunization status^2^	
Fully-immunized	403 (58.7)
Partially-immunized	187 (27.6)
Never-immunized	95 (13.7)
Vitamin A uptake^3^ (Yes), n (%)	421 (61.3)
Caretakers	
Age^4^ (years), median (IQR)	27 (23–33)
Relationship with the child, n (%)	
Mother	639 (93.5)
Father	36 (5.2)
Another family member	9 (1.2)
Unknown	1 (0.1)
Marital status, n (%)	
Married/in a union	654 (95.4)
No partner	29 (4.5)
Unknown	2 (0.1)
In possession of child’s EPI card, n (%)	399 (58.3)
Educational level, n (%)	
No formal education	288 (41.9)
Primary	261 (38.0)
Secondary or above	135 (20.0)
Unknown	1 (0.1)
Source of income, n (%)	
Formal employment	32 (4.3)
Self-employed	608 (89.0)
No income	43 (6.6)
Unknown	2 (0.1)
Literacy, n (%)	
Fully literate	143 (21.2)
Partially literate	189 (27.5)
Illiterate	353 (51.3)
Religion, n (%)	
Christian	409 (59.5)
Animist	211 (31.0)
Other^5^	29 (3.9)
No religion	33 (5.4)
Unknown	3 (0.2)
Ethnic group, n (%)	
Adja	241 (35.1)
Ewe	151 (22.1)
Kabye	149 (21.6)
Lamba	55 (8.1)
Other	88 (13.0)
Unknown	1 (0.1)
Household heads	
Relationship with the child, n (%)	
Father	529 (77.1)
Mother	83 (12.3)
Another family member^6^	73 (10.6)
Educational level, n (%)	
No formal education	263 (38.4)
Primary	239 (34.8)
Secondary or above	183 (26.8)
Source of income, n (%)	
Formal employment	37 (5.3)
Self-employed	600 (87.9)
No income	48 (6.9)
Households	
Number of U5^7^ living in the household, n (%)	
1	427 (63.2)
2	225 (32.2)
3–4	33 (4.6)
Distance from the village to the nearest health facility, n (%)	
< 5 km	301 (43.9)
5–9 km	156 (26.3)
10–19 km	180 (22.8)
≥ 20 km	48 (7.0)

^1^IQR: interquartile range

^2^Information obtained from child’s EPI card (58%) and on the basis of caretaker’s self-declaration (42%)

^3^Recommended for children at 9 months of age

^4^n = 11 with unknown age

^5^Islam: n = 27, Hindu: n = 2

^6^Grandfather, grandmother, uncle, aunt, and other

^7^U5: children under 5 years of age

Caretakers were mostly mothers (93.5%) and had a median age of 27 years (IQR: 23–33). Almost all declared being married or in a relationship (95.4%). The majority were self-employed (89.0%). They had low education levels (41.9% with no formal education and 38.0% at primary level), and few were fully literate (21.2%). Around 60% were Christians, and 31.0% were animists. Only 58.3% declared being in possession of the child’s EPI card.

In 77.1% of the households, the household head was the child’s father. Only 26.8% of the household heads had secondary or higher education, and the majority (87.9%) were self-employed. In more than 60% of the households, the child participant was the only U5 child.

### Caretakers’ knowledge and perceptions regarding malaria

The caretakers had a good knowledge about malaria (94.7%), its cause, and how to prevent it (Table 2). More than 90% believed that the disease was caused by mosquito bites and considered that it could be serious for children. The great majority (87.8%) recognized that sleeping under a bed net was an effective way to prevent the disease. Overall, 95% of caretakers declared that they would like their child to take preventive drugs against malaria.

**Table 2:** Caretakers’ knowledge and perception about malaria (N = 685)

	n (weighted %)
Knowledge	
Know malaria (Yes)	648 (94.7)
Cause of malaria (N = 685)	
Mosquito bites	639 (93.3)
Hot weather and sun	187 (27.2)
Stagnant water	132 (19.2)
Dirty house	73 (10.6)
Bad food	24 (3.4)
Dissatisfaction of ancestors or spirits	9 (1.3)
Family conflict	4 (0.6)
I don't know	34 (4.9)
How to avoid malaria in infants (N = 685)	
Sleeping under a bed net	601 (87.8)
Cleaning up the house	184 (73.1)
Taking drugs	96 (14.0)
Draining stagnant water	62 (9.1)
Using traditional plants	43 (6.4)
Keeping harmony in the community	37 (5.5)
Respecting ancestor’s law	14 (2.1)
Cannot avoid it, it’s our destiny	2 (0.3)
I don’t know	36 (5.1)
Perception	
Malaria can be serious for infants	
Agree	651 (95.1)
Don’t agree/Don’t know	34 (4.9)
I would like my child to take drugs to avoid malaria	
Yes	654 (95.4)
No	8 (1.2)
I don’t know	23 (3.4)

### Prevalence of malaria infection and clinical malaria

**Fig. 2:**
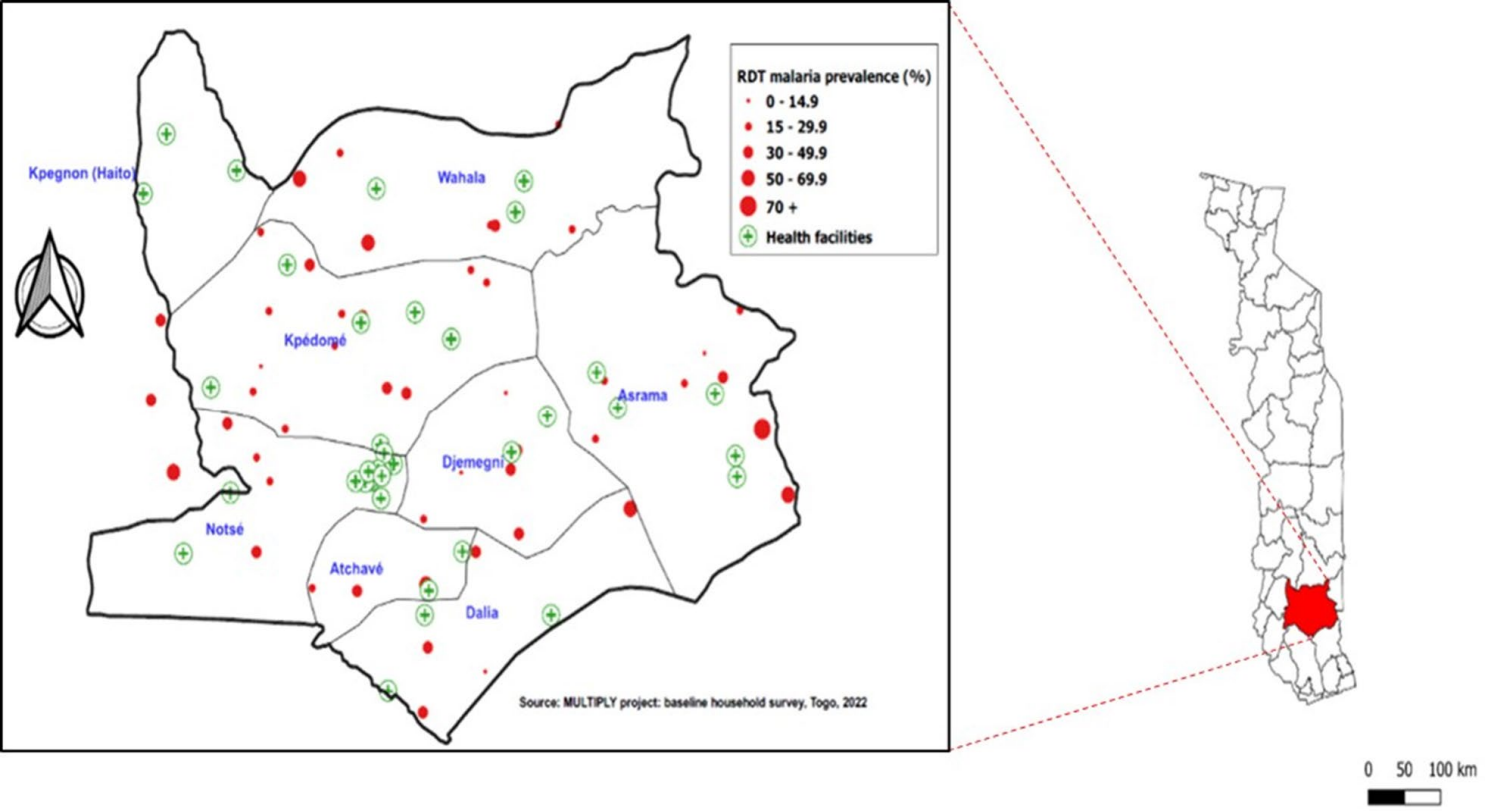
Prevalence of *Plasmodium* infection (RDT positive) in Haho district, Plateaux region, Togo

The prevalence of malaria infection was 32.1% (95% CI 27.7–37.0), with significant variation across areas (cluster range: 0.0–73.3) (Fig. 2). Out of 57 clusters, 14 had a prevalence higher than 50%, of which 5 reached almost 70%. Of the 220 children with a positive RDT, 107 had a fever in the last 24 h or on the day of the survey (temperature ≥ 37.5 °C), yielding a prevalence of clinical malaria of 15.4% (95% CI 12.2–19.2).

### Factors associated with malaria infection

As Table 3 shows, the results of the first multivariable regression model have shown that malaria infection was not associated with any of the child’s characteristics including whether or not the child has slept under a mosquito net the night before (adjusted odds ratio [aOR] 1.27, 95% CI 0.88–1.83). Older children (17–23 months of age) were not at a lower risk of malaria compared to those age 10–16 months (aOR 0.74, 95% CI 0.49–1.11). In the second model, children with caretakers who had animist beliefs had a higher risk of malaria infection (aOR 1.72, 95% CI 1.15–2.56). Children in the households where mothers were the household head were at a significantly higher risk of malaria infection (aOR: 2.51, 95% CI 1.39–4.56).

**Table 3:** Risk factors associated with *Plasmodium* infection in U2 in Haho district (N = 685)

Characteristic	No. of children with malaria infection (%)	Univariate			Model 1 (Child model)^a^		Model 2 (Caretaker & household head model)^a^				Model 3 (Household model)^a^		
		OR	95 CI	p	aOR	95 CI	p	aOR	95 CI	p	aOR	95 CI	p
Child’s characteristics													
Age				0.17			0.14			0.23			0.21
10–16 months	87/302 (28.8)	Ref	–		–	–		–	–		–	–	
17–23 months	133/383 (34.7)	0.76	0.50, 1.14		0.74	0.49, 1.11		0.76	0.48, 1.21		0.76	0.49, 1.18	
Sleeping under a bed net the night before				0.19			0.25						
Yes	168/541 (31.1)	–	–		–	–							
No	52/144 (36.1)	1.27	0.88, 1.83		1.24	0.85, 1.81							
Immunization status				0.26			0.93						
Fully immunized	117/403 (29.0)	–	–		–	–							
Partially immunized	66/187 (35.3)	1.51	0.80, 2.85		1.14	0.47, 2.73							
Not immunized	37/95 (38.9)	1.33	0.85, 2.10		1.12	0.59, 2.13							
Vitamin A uptake				0.11			0.50						
Yes	122/421 (29.0)	–	–		–	–							
No	98/264 (37.1)	1.43	0.91, 2.25		1.29	0.61, 2.73							
Caretaker’s characteristics													
Age				0.67						0.49			
< 27 years	94/300 (31.3)	–	–					–	–				
$\ge$ 27 years	126/385 (32.7)	1.08	0.75, 1.54					0.88	0.60, 1.29				
Marital status				0.99						0.10			
Married/in union	210/654 (32.1)	–	–					–	–				
No partner	10/31 (32.3)	1.01	0.44, 2.31					0.49	0.20, 1.18				
Educational level				0.024						0.49			
Secondary or above	31/135 (23.0)	–	–					–	–				
Primary	84/261 (32.2)	1.57	1.01, 2.44					0.92	0.46, 1.83				
No formal education	105/288 (36.3)	1.95	1.19, 3.18					1.32	0.46, 1.83				
Source of income				0.48						0.39			
Formally-employed	12/32 (37.5)	–	–					–	–				
Self-employed	190/608 (31.2)	0.81	0.43, 1.52					0.76	0.32, 1.80				
No income	18/45 (40.0)	1.18	0.49, 2.83					1.24	0.47, 3.26				
Literacy				0.092						0.20			
Fully literate	33/143 (23.1)	–	–					–	–				
Partially literate	67/189(35.4)	1.81	1.03, 3.18					1.43	0.68, 3.01				
Illiterate	120/353 (34.0)	1.73	1.00, 3.01					0.91	0.38, 2.18				
Religion				< 0.001						0.004			0.001
Christian	115/409 (28.1)	–	–					–	–		–	–	
Animist	89/211 (42.2)	1.91	1.33, 2.73					1.72	1.15, 2.56		1.71	1.19, 2.46	
Other	16/65(24.6)	0.86	0.50, 1.48					0.82	0.42, 1.62		0.75	0.41, 1.37	
Ethnic group				0.13						0.064			
Ewe	47/151 (31.1)	–	–					–	–				
Adja	85/241 (35.3)	1.21	0.71, 2.08					0.96	0.53, 1.73				
Kabve	40/149 (26.8)	0.84	0.44, 1.60					0.75	0.38, 1.50				
Lamba	24/55 (43.6)	1.73	0.90, 3.33					1.88	0.92, 3.84				
Other/unknown	24/89 (27.0)	0.81	0.42, 1.56					0.79	0.38, 1.65				
Possession of child’s EPI card				0.62						0.65			
Yes	124/399 (31.1)	–	–					–	–				
No	96/286 (33.6)	1.10	0.76, 1.59					0.91	0.60, 1.38				
Household head’s characteristics													
Relationship with the child				< 0.001						< 0.001			< 0.001
Father	160/529 (30.2)	–	–					–	–		–	–	
Mother	43/83 (51.8)	2.47	1.50, 4.07					2.51	1.39, 4.56		2.39	1.43, 3.99	
Another family member	17/73 (23.3)	0.68	0.40, 1.15					0.58	0.34, 0.99		0.63	0.37, 1.06	
Educational level				< 0.001						0.18			
Secondary or above	39/183 (21.3)	–	–					–	–				
Primary	84/239 (35.1)	2.04	1.32, 3.15					1.60	0.93, 2.74				
No formal education	97/263 (36.9)	2.17	1.36, 3.46					1.57	0.84, 2.93				
Source of income				0.014						0.43			
Formal employment	6/37 (16.2)	–	–					–	–				
Self-employed	192/600 (32.0)	2.73	1.04, 7.14					1.85	0.71, 4.83				
No income	22/48 (45.8)	4.85	1.62, 14.6					1.76	0.49, 6.30				
Household’s characteristics													
Number of U5 living in the household				0.038									0.040
1	124/427 (29.0)	–	–								–	–	
≥ 2	96/258 (37.2)	1.46	1.01, 2.11								1.44	1.01, 2.07	
Distance from the village to the nearest health facility				0.008									0.027
< 5 km	77/301 (25.6)	–	–								–	–	
≥ 5 km	143/384 (37.2)	1.75	1.15, 2.66								1.60	1.04, 2.44	

^a^Clusters included as a random effect

*OR* odds ratio, *CI* confidence interval, *aOR* adjusted odds ratio, *U5* children under 5 years of age

In the third and final model, household characteristics were assessed in addition to the child’s age and other two variables that showed significant association in the previous model (caretaker’s religion and the type of household head). Village location was found to be a significant predictor of children’s infection status. When the village was located more than 5 km away from the nearest health facility, the children were more likely to have a positive RDT (aOR: 1.60, 95% CI 1.04–2.44). Also, children living in a household where there were two or more children U5 were at a significantly higher risk of infection (aOR: 1.44, 95% CI 1.01–2.07). Association with the caretaker’s religion and the type of household head remained significant in the final model (aOR: 1.71, 95% CI 1.19–2.46, aOR: 2.39, 95% CI 1.43–3.99, respectively), whereas children’s age showed no association (aOR: 0.76, 95% CI 0.49–1.18).

## Discussion

The prevalence of *Plasmodium* infection was 32.1% among children in the survey with significant heterogeneity across areas. One-half of them had clinical malaria (15.4%). High prevalence was observed in certain parts of the district. This study is the first to report such detailed sub-district-level data on malaria prevalence in this area.

The 2020 national survey reported a prevalence of 48.7% in the Plateaux region, measured in November and December by microscopy [3]. While the results of these two surveys are not directly comparable due to the differences in seasons, survey methods, areas, and population (the national survey included children 6–59 months of age), the prevalence found in the study is striking given that one in three children U2 had a positive RDT in the dry season despite caretakers’ good knowledge and high bed net use. The high prevalence of malaria infection might be a reflection of malaria case resurgence in Togo and elsewhere in recent years [1, 28–30].

In the present study, several factors were associated with a child’s infection status, in line with the findings from previous studies. The body of evidence has shown that low socioeconomic status was a significant predictor of malaria [19, 20, 24–26, 31]. The results from the current study confirmed that children in mother-headed households were at a higher risk of malaria. Due to lack of information, socioeconomic status of mother-headed households was not measured precisely. Nonetheless, the proportion of household heads who had no income was found to be significantly higher in the mother-headed households than those headed by a father or another family member (26.5%, 4.3%, 4.1% respectively, P < 0.001). Likewise, the proportion of household heads with no formal education was significantly higher in the former than the father-headed households (51.8% vs. 33.6%, P < 0.001). A previous study in Togo reported that malaria-related expenditure accounted for about 5% of monthly household spending and that female-headed households were more likely to fall into the situation of “catastrophic health expenditure” corresponding to 10% of total household spending [32, 33]. It is thus reasonable to assume that the children in mother-headed households were more likely to be exposed to higher infection risk due to sub-optimal prevention efforts and underlying poor living conditions. The increased risk faced by the children living in a household with multiple young children might also indicate family hardship in meeting the needs of young siblings. An alternative explanation might be that household population density, a known risk factor for malaria, might have increased malaria risk in children living in crowded households; however, the actual size of the households was not known in the present study, making it difficult to draw a firm conclusion [22, 26].

Children living in a cluster located more than 5 km away from a health facility were at increased risk of malaria infection. In Togo, in order to reach the population living in such remote areas, CHWs are deployed to relay essential malaria-related services including diagnosis and treatment of uncomplicated cases with artemisinin-based combination therapy. Routine EPI services are also delivered through outreach activities together with other mother-and-child health services including antenatal care, preventive treatment of malaria for pregnant women, and distribution of bed nets to pregnant women and children under 12 months of age. Further investigations of the areas where extremely high prevalence was observed (14 clusters where prevalence was ≥ 50%) found that almost one-half were located more than 10 km away from the nearest health facility. Of these, half were a distance greater than 20 km. While such high prevalence can be explained by reduced access to healthcare and information, it might also be the consequences of environmental risk factors including land use for farming, presence of livestock, proximity to breeding sites, and housing quality, which could not be untangled due to lack of information. Nonetheless, the findings suggest that more concerted efforts are needed to address the vulnerability of children living in such hard-to-reach areas. The current efforts of the Togolese Ministry of Health to integrate CHWs more formally into the health system should thus be seen as a positive step towards improving health equity and reducing the burden of malaria in these high transmission areas.

Lack of association between a child’s infection outcome and bed net use has been reported previously [22, 25, 31, 34]. Recently, some studies in Togo have shown evidence of high insecticide resistance in *Anopheles gambiae *sensu lato (*s.l.*), the major malaria vector in the country, particularly to dichlorodiphenyltrichloroethane and pyrethroids [35–37]. This might offer partial explanation to the seemingly insufficient effectiveness of the bed nets currently in use in the area; however, evidence of insecticide resistance rendering bed nets ineffective is not conclusive in the literature [38]. The finding therefore warrants caution for interpretation. As stated earlier, the information about children’s bed net use was given by caretakers themselves, which might have somewhat compromised the information’s validity. Furthermore, no information was available to investigate the bed nets’ condition or their proper or consistent usage. The national survey in 2020 showed that household ownership of LLINs has been steadily increasing, but its utilization in children U5 has stagnated at around 70% since 2017 [30]. Despite the limits of the data and the complexity of interpretation, the findings from the present study still highlight the need to reinforce the malaria prevention strategy through a combination of different tools, as recommended by the WHO [12].

An alternative prevention strategy such as PMC can further strengthen existing efforts and contribute significantly to reducing malaria burden in the area. The caretakers’ high acceptability of preventive drugs, as demonstrated in the survey, is encouraging and will greatly facilitate the introduction of PMC. In Sierra Leone, the only country where malaria chemoprevention has been implemented as part of the national strategy in perennial transmission settings, children U2 who have received three doses of SP were less likely to have malaria infection (aOR 0.62, 95% CI 0.38–1.02) [34]. The present study has shown that one-half of children who tested positive for RDT were asymptomatic while forming a reservoir for transmission. Introduction of PMC can contribute to reducing the malaria reservoir in the community if sufficient coverage is achieved. The current PMC scheme tested in the Haho district corresponds with the present EPI schedule in Togo. This means that the last dose of SP is administered to children at the age of 15 months (at the visit of the second measles vaccination), which will protect them for the following 28 days [5]. The present analysis did not demonstrate a lower risk of malaria infection in children age 17–23 months compared to those age 10–16 months. The NMCP could seek other contact points for PMC to better protect children in the second year of life, in accordance with the recent recommendations made by the WHO [12].

## Conclusion

This study provided information on the local malaria epidemiology in the Haho district before the introduction of pilot PMC implementation and identified risk factors to guide more effective malaria control measures in the area. The malaria prevalence was 32% with significant area variation. Coupled with the existing preventive strategy, PMC can play an important role in reducing malaria burden in this high transmission setting. To better protect children in the second year of life, the NMCP might consider means other than the routine EPI platform to deliver PMC. Delivering an effective malaria preventive strategy should be viewed within a larger framework of interventions that aim to improve the socioeconomic status of poor households. Efforts to reach children living in some hard-to-reach areas should continue and be strengthened in order to reduce inequity in their health outcome.

## Data Availability

The dataset supporting the conclusions of this article is available in CORA.RDR (Catalan Open Research Area. Repositori de dades de Recerca) repository, https://doi.org/10.34810/data887.
